# Critical Role of the Interaction Gut Microbiota – Sympathetic Nervous System in the Regulation of Blood Pressure

**DOI:** 10.3389/fphys.2019.00231

**Published:** 2019-03-08

**Authors:** Marta Toral, Iñaki Robles-Vera, Néstor de la Visitación, Miguel Romero, Tao Yang, Manuel Sánchez, Manuel Gómez-Guzmán, Rosario Jiménez, Mohan K. Raizada, Juan Duarte

**Affiliations:** ^1^Department of Pharmacology, School of Pharmacy, Centro de Investigación Biomédica, University of Granada, Granada, Spain; ^2^Instituto de Investigación Biosanitaria de Granada, ibs.GRANADA, Granada, Spain; ^3^Department of Physiology and Functional Genomics, University of Florida, Gainesville, FL, United States; ^4^CIBERCV, University of Granada, Granada, Spain

**Keywords:** gut dysbiosis, hypertension, oxidative stress, neuroinflammation, sympathetic nervous system

## Abstract

Association between gut dysbiosis and neurogenic diseases, such as hypertension, has been described. The aim of this study was to investigate whether changes in the gut microbiota alter gut-brain interactions inducing changes in blood pressure (BP). Recipient normotensive Wistar-Kyoto (WKY) and spontaneously hypertensive rats (SHR) were orally gavaged with donor fecal contents from SHR and WKY. We divided the animals into four groups: WKY transplanted with WKY microbiota (W-W), SHR with SHR (S-S), WKY with SHR (W-S) and SHR with WKY (S-W). Basal systolic BP (SBP) and diastolic BP (DBP) were reduced with no change in heart rate as a result of fecal microbiota transplantation (FMT) from WKY rats to SHR. Similarly, FMT from SHR to WKY increased basal SBP and DBP. Increases in both NADPH oxidase-driven reactive oxygen species production and proinflammatory cytokines in brain paraventricular nucleus linked to higher BP drop with pentolinium and plasmatic noradrenaline (NA) levels were found in the S-S group as compared to the W-W group. These parameters were reduced by FMT from WKY to SHR. Increased levels of pro-inflammatory cytokines, tyrosine hydroxylase mRNA levels and NA content in the proximal colon, whereas reduced mRNA levels of gap junction proteins, were found in the S-S group as compared to the W-W group. These changes were inhibited by FMT from WKY to SHR. According to our correlation analyses, the abundance of *Blautia* and *Odoribacter* showed a negative correlation with high SBP. In conclusion, in SHR gut microbiota is an important factor involved in BP control, at least in part, as consequence of its effect on neuroinflammation and the sympathetic nervous system activity.

## Introduction

Abundant evidence has demonstrated the association between gut dysbiosis and neurogenic diseases, such as hypertension (Mell et al., 2015; Yang et al., 2015). A common characteristic of resistant hypertension is chronically elevated sympathetic nervous system (SNS) activity accompanied by a high release of noradrenaline (NA) (Tsioufis et al., 2011), which indicates a neurogenic component that contributes to the initiation, maintenance and progression of hypertension (Yang and Zubcevic, 2017).

The factors that stimulate sympathetic tone in human essential hypertension are poorly understood. A newly identified interaction between the brain, gut and bone has been identified as a possible mechanism in the pathogenesis of hypertension (Santisteban et al., 2016). For instance, an increase in sympathetic drive to bone marrow (BM) and the gut may also trigger a sequence of signaling events that can, ultimately, contribute to an overall increase in blood pressure (BP) and the establishment of hypertension, by affecting the structure and function of BP target organs, such as vasculature, kidney, heart, and brain. Increased sympathetic activity to the gut could result in dysbiosis, increased gut permeability and inflammatory status, leading to an imbalance in the gut content of short-chain fatty acids (SCFAs)-producing bacteria and in the plasma levels of lipopolysaccharide (LPS). These metabolic and structural microbial products, working together, elevate sympathetic drive to the BM and other lymphoid organs, and may act as modulators for BM cell activity by increasing the proliferation and release of myeloid progenitors and other pro-inflammatory cells. This increase in myeloid progenitor cells contributes to an increase in peripheral and central inflammation that could be a critical event for the establishment of hypertension.

The immune and sympathetic systems are recognized to contribute to the development of hypertension (Yang et al., 2017), while there exists a bidirectional signaling between the brain and gut microbiota which can regulate the BP through the modulation of the interaction between SNS and immune system (Yang and Zubcevic, 2017). Therefore, since a link between hypertension and gut dysbiosis has recently been suspected (Mell et al., 2015; Yang et al., 2015), several groups are investigating this interaction. In this way, different studies have shown that fecal microbiota transplantation (FMT) from hypertensive human and rat donors elevate the BP of the host, normotensive mice and rats, respectively (Adnan et al., 2017; Li et al., 2017; Toral et al., 2018), pointing out that gut dysbiosis plays a possible contributing or even causal role in hypertension. However, the communication between gut microbiota and the SNS in hypertension is not completely understood. We hypothesize that an increased sympathetic activity contributes to gut dysbiosis, which then increases inflammation and sympathetic activity. Because of this, in the present study, we tested whether changes in gut microbiota composition induced by reciprocal fecal microbiota transplantation from Wistar-Kyoto (WKY) to spontaneously hypertensive rats (SHR), could decrease neuroinflammation and sympathetic activity and thereby lower BP. We used SHR as a model of neurogenic hypertension characterized by sustained age-dependent elevation in sympathetic activity and dysbiosis (Judy et al., 1976; Yang and Zubcevic, 2017).

## Materials and Methods

### Animals and Experimental Groups

This research was performed according to the National Institutes of Health (NIH) Guide for the Care and Use of Laboratory Animals, and approved by the Ethic Committee of Laboratory Animals of the University of Granada, Spain (Ref. 03-CEEA-OH-2013). Male SHR and WKY were obtained from Harlan Laboratories (Barcelona, Spain). All rats were fed standard rat chow (Harlan global diet 2014, Harlan Laboratories, Inc., Milan, Italy) *ad libitum* for the duration of the experiment. Stool samples were collected and pooled from twenty-week-old WKY and SHR rats. Donor fecal contents were administered through oral gavage to twenty-five-weeks-old WKY and SHR rats for 3 consecutive days, and once every 3 days for a total extension of 4 weeks. Animals were randomly assigned to four different groups of 5–8 animals each: WKY with WKY microbiota (W-W), WKY with SHR (W-S), SHR with SHR (S-S) and SHR with WKY (S-W). Rats were kept in individually ventilated cages in a pathogen-free animal facility. Body weight, food and water intake were recorded weekly for all groups. During the experimental periods, rats had free access to tap water and chow. FMT to recipient rats were carried out as previously reported with several modifications (Bruce-Keller et al., 2015).

### Fecal Microbiota Transplantation (FMT)

Fecal icrobiota transplantation to recipient rats was carried out as previously reported with several modifications (Toral et al., 2018). Briefly, fecal contents were isolated and pooled from WKY rats and SHR (*n* = 5). Fecal contents were diluted 1:20 in sterile PBS and centrifuged at 800 rpm for 5 min. The supernatant was aliquoted and stored at -80°C. Starting 1 week before the administration, recipient rats were administered with 1 mL ceftriaxone sodium (400 mg/Kg/day) daily for five consecutive days by oral gavage. The purpose of the antibiotic treatment was to reduce the pre-existing microbiota and to facilitate the recovery of the population and diversity of intestinal microbiota from donor rats after FMT (Li et al., 2017). Forty-eight hours after the last antibiotic treatment, recipient rats were orally gavaged with donor fecal contents (1 mL) as explained above.

### Blood Pressure Measurements

Systolic blood pressure (SBP) and heart rate (HR) was measured weekly at room temperature using tail-cuff plethysmography as described previously (Zarzuelo et al., 2011). At the end of the experimental period, animals were subjected to isoflurane anesthesia, a polyethylene catheter containing 100U heparin in isotonic, sterile NaCl solution was inserted in the left carotid artery to monitor intra-arterial BP. Twenty-four hours after the implantation of the catheter, we recorded intra-arterial BP uninterruptedly for 60 min with a sampling frequency of 400/s (McLab; AD Instruments, Hastings, United Kingdom). For intergroup comparisons, BP values recorded during the last 30 min were averaged.

### Evaluation of the Contribution of Sympathetic Activity

Acute BP responses to intravenous injection of pentolinium (10 mg/Kg) were analyzed in conscious rats. Before pentolinium administration, arterial blood samples (0.2 mL) were drawn via the catheter to measure NA levels and plasma renin activity (PRA). The pentolinium dose was selected because it produces maximal sympathetic inhibition (Pechánová et al., 2004). Finally, the rats were subjected to isoflurane anesthesia and were killed by complete exsanguination, then the brain was removed, snap-frozen in liquid nitrogen, and stored at -80°C until processed for the reverse transcriptase-polymerase chain reaction (RT-PCR) measurement (Romero et al., 2016).

### Plasma and Colonic Determinations

Blood samples were cooled in ice and centrifuged for 10 min at 3,500 rpm at 4°C, and the plasma was frozen at -80°C. Plasma LPS concentration was measured using the Limulus Amebocyte Lyste (LAL) chromogenic endotoxin quantitation Kit (Lonza, Valais, Switzerland), according to the instructions of the manufacturer.

We used enzyme-linked immunosorbent assay kits (IBL International, Hamburg, Germany) to measure both plasma and colonic NA concentrations following the manufacturer’s protocol. Colon samples were collected and immersed in the appropriate conservation solution. EDTA 1 mM and sodium metabisulfite 4 mM were added to prevent the catecholamine degradation, then plasma and tissue samples were stored at -80°C for later use.

The Rat Renin Activity Fluorometric Assay Kit (BioVision, Milpitas, CA, United States; K806-100) was used to measure the renin activity following the manufacturer’s protocol.

### Measurement of Intracellular Reactive Oxygen Species (ROS) Concentrations

Reactive oxygen species production was measured in homogenates from brain paraventricular nucleus (PVN) using the fluorescent probe 5-(and-6-)chloromethyl-2′-7′-dichlorodihydrofluorescein diacetate (CM-H2DCFDA). Brain PVN was homogenized in lysis buffer composed of 50 mM Tris–HCl (pH 7.4) containing 0.1 mM EDTA, 0.1 mM EGTA, 10 μg/mL aprotinin, 10 μg/mL leupeptin and 1 mM PMSF. Fresh homogenates (10 μg of protein) in 96-well plates were incubated with 5 μmol/L CM-H2DCFDA for 30 min at 37°C, in the absence or in the presence of the NADPH oxidase inhibitor apocynin (50 μM). The fluorescent intensity was measured using a spectrofluorimeter (Fluorostart, BMG Labtech, Ortenberg, Germany) (Toral et al., 2015).

### NADPH Oxidase Activity

The NADPH oxidase activity in homogenates from brain PVN was measured by dihydroethidium (DHE) fluorescence assay in the microplate reader, as described previously (Fernandes et al., 2007; Toral et al., 2015). Fresh homogenates (10 μg of protein) were incubated with DHE (10 μM) and deoxyribonucleic acid (DNA, 1.25 μg/mL) in PBS (100 mM), pH 7.4, containing 100 μM DTPA with the addition of NADPH (50 μM), at a final volume of 120 μL. Incubations were performed for 30 min at 37°C in the dark. Total fluorescence was followed in a microplate reader using a rhodamine filter (excitation 490 nm and emission 590 nm) in a spectrofluorometer (Fluorostart, BMG Labtech, Ortenberg, Germany).

### RT-PCR Analysis

For RT-PCR analysis, total RNA was extracted from the colon, and brain PVN by homogenization and converted to cDNA by standard methods. PVN and colon tissue was homogenized in 1 ml of TRI Reagent (Thermo Fisher Scientific Inc., Waltham, MA, United States). RNA isolation was performed with traditional methods using sequential washes with bromochloropropane, isopropanol and ethanol 75%. RNA concentrations were measured with a NanoDrop^TM^ 2000 Spectrophotometer (Thermo Fisher Scientific Inc., Waltham, MA, United States). A Techne Techgene thermocycler (Techne, Cambridge, United Kingdom) was used to perform the polymerase chain reaction. mRNA expression was analyzed through quantitative real-time RT-PCR. RNA was reverse transcribed using oligo (dT) primers, Recombinant RNasin^®^ Ribonuclease, dNTP (10 mM) and M-MLV reverse Transcriptase (Promega, Southampton, United Kingdom). Reverse resulting cDNA (2 ng) was amplified on optical grade 48-well plates in an Eco^TM^ Real-Time PCR System (Illumina, CA, United States), using GoTaq^®^ qPCR Master Mix, 2x (Promega, Southampton, United Kingdom). In Table 1 are listed the sequences of both sense and antisense primers used for amplification. In order to determine non-saturating conditions of PCR amplification for all genes studied, preliminary experiments with various amounts of cDNA were performed. Under these conditions, RT-PCR method was used to assess the relative quantification of mRNA. A standard tissue sample was used to determine the efficiency of the PCR reaction. The ΔΔCt method was performed for quantification. The housekeeping gen glyceraldehyde-3-phosphate dehydrogenase (GAPDH) was used for internal normalization (Romero et al., 2016).

**Table 1:** Oligonucleotides for real-time RT-PCR.

mRNA targets	Descriptions	Sense	Antisense
NOX-1	NOX-1 subunit of NADPH oxidase	TCTTGCTGGTTGACACTTGC	TATGGGAGTGGGAATCTTGG
NOX-4	NOX-4 subunit of NADPH oxidase	ACAGTCCTGGCTTACCTTCG	TTCTGGGATCCTCATTCTGG
p22phox	p22phox subunit of NADPH oxidase	GCGGTGTGGACAGAAGTACC	CTTGGGTTTAGGCTCAATGG
p47phox	p47phox subunit of NADPH oxidase	CCCAGCGACAGATTAGAAGC	TGGATTGTCCTTTGAGTCAGG
TNF-α	Tumor necrosis factor-alpha	ACGATGCTCAGAAACACACG	CAGTCTGGGAAGCTCTGAGG
IL-6	Interleukin-6	GATGGATGCTTCCAAACTGG	AGGAGAGCATTGGAAGTTGG
IL-10	Interleukin-10	GAATTCCCTGGGAGAGAAGC	GCTCCACTGCCTTGCTTTTA
IL-17a	Interleukin-17a	CTTCACCTTGGACTCTGAGC	TGGCGGACAATAGAGGAAAC
IFNγ	Interferon gamma	GCCCTCTCTGGCTGTTACTG	CCAAGAGGAGGCTCTTTCCT
cd11b	Cd11b	GAGAACTGGTTCTGGCTTGC	TCAGTTCGAGCCTTCTT
CCL2	C-C chemokine ligand 2	CCTCCACCACTATGCAGGTC	CAGCCGACTCATTGGGATCA
Olfr59	Olfactory receptor 59	CTGCTAGTCATGGGTGTAGATG	CAAGGGTGATAGAACGGTAAGG
GPR-41	G-protein-coupled receptor-41	TGACGGTGAGCATAGAACGTTT	GCCGGGTTTTGTACCACAGT
GPR-43	G-protein-coupled receptor-43	TCGTGGAAGCTGCATCCA	GCGCGCACACGATCTTT
Occludin	Occludin	AGCCTGGGCAGTCGGGTTGA	ACACAGACCCCAGAGCGGCA
Muc2	Mucin-2	CGATCACCACCATTGCCACTG	ACCACCATTACCACCACCTCAG
ZO-1	Zonula occludens-1	GCCAGCCAGTTCCGCCTCTG	AGGGTCCCGGGTTGGTG
IL-1β	Interleukin-1 beta	GTCACTCATTGTGGCTGTGG	GCAGTGCAGCTGTCTAATGG
TH	Tyrosine hydroxylase	GATTGCTACCTGGAAGGAGGT	AGTCCAATGTCCTGGGAGAAC
GAPDH	Glyceraldehyde-3-Phosphate Dehydrogenase	ACCACAGTCCATGCCATCAC	TCCACCACCCTGTTGCTGTA

### 16S rDNA V4-V5 Region Sequencing

Fecal DNA was extracted from the samples collected from 5 to 6 animals per group by using a quick-DNA fecal/soil microbe kit (Zymo Research, Irvine, CA). Primers compatible with illumina Miseq v2 2x250bp kit (Illumina, San Diego, CA) were used to amplify bacterial 16S V4–V5 variable regions (Robles-Vera et al., 2018). The PCR amplicons were purified using a QIAquick gel extraction kit (QIAGEN, Hilden, Germany) and quantified by Qubit (thermos Fisher Scientific, Waltham, MA, United States). Equal amounts of purified PCR product from each sample were pooled together as one library. The library was quantified by real time PCR (Kapa Biosystems, Wilmington, MA, United States) prior to Miseq sequencing (Illumina, San Diego, CA, United States). The sequencing data had a Q30 score ≥ 93.5% and 97.17 ± 0.34% of total cluster passes the filter.

### Bioinformatics Analysis

The raw paired-reads from Miseq were processed using QIIME 1.9.1. Briefly, reads were trimmed to remove bases with Phred scores lower than 30 and quality-filtered with parameters set as previously optimized (ref: Quality-filtering vastly improves diversity estimates from Illumina amplicon sequencing). Open reference OTU-picking was performed and taxonomical assignment to the generated OTUs were performed with 97% identity against Greengenes database 13.8. Alpha diversity and unweighted principal coordinate analyses plots using the phylogenic tree-based unifrac distance metric were generated using scripts from QIIME package.

### Reagents

All reagents were purchased from Sigma-Aldrich (Barcelona, Spain) unless otherwise specified.

### Statistical Analysis

Statistical analyses were performed with GraphPad Prism 7 software. Results are expressed as means ± SEM. To test if the values come from a gaussian distribution a Shapiro-Wilk normality test was used. For comparisons of the four groups with two variables (strain and treatment) we used a two-way ANOVA (with Sidak’s correction for comparison of multiple means). Factors were partitioned into strain (WKY-SHR) and FMT (from WKY/from SHR). A *p* value of less than 0.05 was considered significant considering the main effects of the strain (WKY-SHR), FMT (from WKY/from SHR) and their interaction (I; strain vs. FMT).

## Results

### BP Is Controlled by Gut Microbiota

**FIGURE 1:**
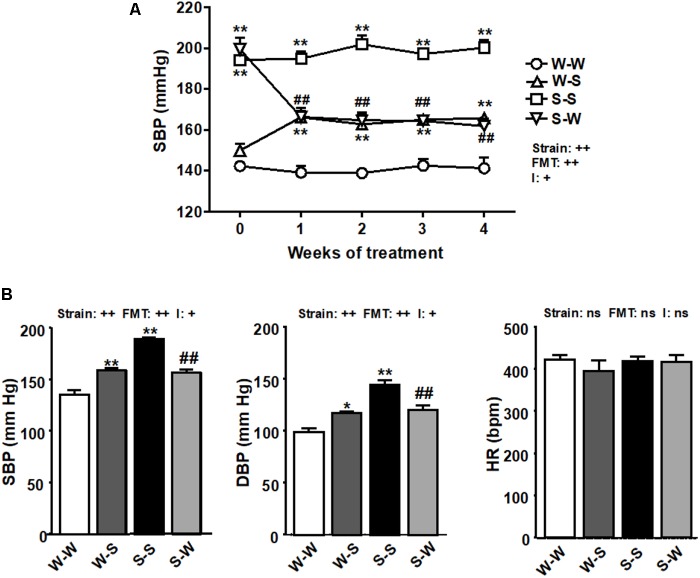
Effects of fecal microbiota transplantation (FMT) on blood pressure. Systolic blood pressure (SBP), measured by tail-cuff plethysmography during 4 weeks of FMT **(A)**, and SBP, diastolic blood pressure (DBP), and heart rate (HR), measured by direct register **(B)**, in spontaneously hypertensive rats (SHR) with stool transplant from SHR (S-S) or from Wistar Kyoto rats (WKY) (S-W) and in WKY with stool transplant from WKY (W-W) or from SHR (W-S) at the end of the experimental period. Strain factor, FMT factor and I interaction between strain and FMT factors. ++*p* < 0.01, +*p* < 0.05 and ns (not significant) for the probability based on a two-way analysis of variance. Values are means ± SEM (*n* = 5–8). ^∗^*P* < 0.05 and ^∗∗^*P* < 0.01 vs. W-W; ^##^*P* < 0.01 vs. S-S statistical significance for the probability based on a Sidak’s correction multiple comparisons test.

Fecal exchange from SHR to WKY enhanced basal SBP (149.8 ± 4 mm Hg) by 16 ± 2 mm Hg (*p*_FMT_ < 0.01), measured by tail-cuff plethysmography. Similarly, FMT from WKY rats to SHR lowered SBP by 38 ± 4 mm Hg (*p*_FMT_< 0.01) the basal (199.5 ± 6 mm Hg, *p*_strain_ < 0.01) (Figure 1A) resulting in a significant strain versus FMT interaction (pi < 0.05). In conscious rats, direct SBP and DBP values were also increased by FMT from SHR to WKY as compared to the W-W group (*p*_FMT_< 0.01), and reduced in SHR after FMT from WKY as compared to FMT from SHR to SHR (*p*_FMT_< 0.01), leading to a significant strain versus FMT interaction (pi < 0.05). No significant changes (*p*_FMT_= 0.62, *p*_strain_ = 0.44) were observed among all experimental groups in HR obtained by direct register (Figure 1B). No interaction was observed between strain and FMT (pi = 0.39).

### Sympathetic Activity, Brain PVN NADPH Oxidase, Inflammation, and Macrophages and T Cells Infiltration Are Regulated by Gut Microbiota

**FIGURE 2:**
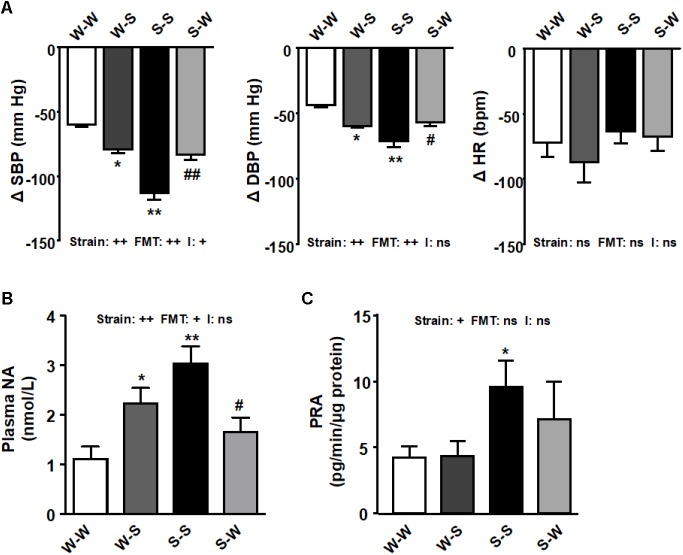
Effects of fecal microbiota transplantation (FMT) on sympathetic tone. Decrease induced by acute intravenous administration of pentolinium (10 mg/kg) on systolic blood pressure (SBP), diastolic blood pressure (DBP), and heart rate (HR), in conscious rats **(A)**. Plasma noradrenaline (NA) levels **(B)**, and plasma renin activity (PRA) **(C)** found in all experimental groups. Strain factor, FMT factor and I interaction between strain and FMT factors. ++*p* < 0.01, +*p* < 0.05 and ns (not significant) for the probability based on a two-way analysis of variance. Values are means ± SEM (*n* = 5–8). ^∗^*P* < 0.05 and ^∗∗^*P* < 0.01 vs. WKY with stool transplant from WKY (W-W); ^#^*P* < 0.05 and ^##^*P* < 0.01 vs. SHR with stool transplant from SHR (S-S), statistical significance for the probability based on a Sidak’s correction multiple comparisons test.

We used a ganglionic blocker, pentolinium, in conscious rats to determine the effect of FMT on sympathetic outflow. The reduction on SBP after ganglionic blockade was higher in the S-S group as compared to the W-W group (-113.3 ± 5.3 mm Hg vs. -59.8 ± 2.5 mm Hg, *p*_strain_ < 0.01). FMT from WKY to SHR inhibited of the decay in SBP after pentolinium (-83.6 ± 4.1 mm Hg, *p*_FMT_< 0.01) as compared to the S-S group, whereas in W-S rats this reduction was also higher (-79.4 ± 3.6 mm Hg, *p*_FMT_< 0.05) than that found in the W-W group (Figure 2A), which led to a significant strain versus FMT interaction (pi < 0.05). Similar qualitative changes among groups in DBP reductions after pentolinium injection were observed (*p*_strain_ < 0.01, *p*_FMT_< 0.01), without interaction between strain and FMT (pi = 0.83). No changes in HR reductions (*p*_FMT_= 0.24, *p*_strain_ = 0.63) and no interaction were observed between strain and FMT (pi = 0.42) (Figure 2A). Plasma NA concentration (*p*_strain_ < 0.01), another marker of sympathetic nerve activity, was reduced ≈ 65 % by FMT from WKY to SHR (*p*_FMT_ < 0.05) and increased ≈ 2.5 times by FMT from SHR to WKY rats (*p*_FMT_ < 0.05) (Figure 2B). PRA was found ≈ 2 times higher in the S-S groups as compared to the W-W group (*p*_strain_ < 0.05). However, neither FMT from SHR to WKY increased PRA, as compared with W-W, nor FMT from WKY significantly reduced PRA as compared with S-S group (*p*_FMT_= 0.52) (Figure 2C), which not led to a significant strain versus FMT interaction (NA concentration: pi = 0.46; PRA: pi = 0.55).

**FIGURE 3:**
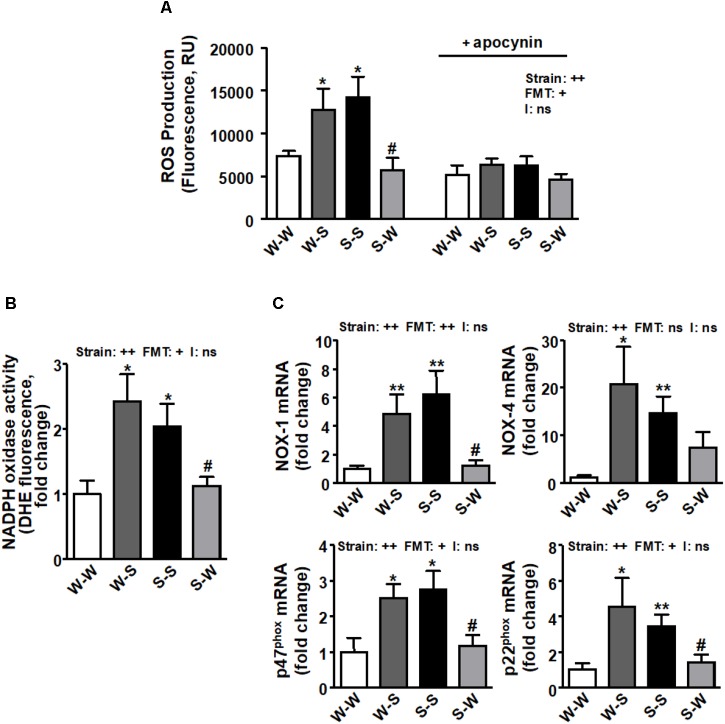
Effects of fecal microbiota transplantation (FMT) on ROS production and NADPH oxidase pathway in the brain PVN. CM-H2DCFDA-detected intracellular ROS in absence and presence of NADPH oxidase inhibitor apocynin (50 μM) **(A)** and NADPH oxidase activity measured by DHE fluorescence measured in the microplate reader **(B)** in homogenates from brain PVN. mRNA levels of NADPH oxidase subunits NOX-1, NOX-4, p47^phox^ and p22^phox^ **(C)** in the brain PVN from all experimental groups. Strain factor, FMT factor and I interaction between strain and FMT factors. ++*p* < 0.01 and ns (not significant) for the probability based on a two-way analysis of variance. Values are means ± SEM (*n* = 5–8). ^∗^*P* < 0.05 and ^∗∗^*P* < 0.01 vs. WKY with stool transplant from WKY (W-W); ^#^*P* < 0.05 vs. SHR with stool transplant from SHR (S-S), statistical significance for the probability based on a Sidak’s correction multiple comparisons test.

**FIGURE 4:**
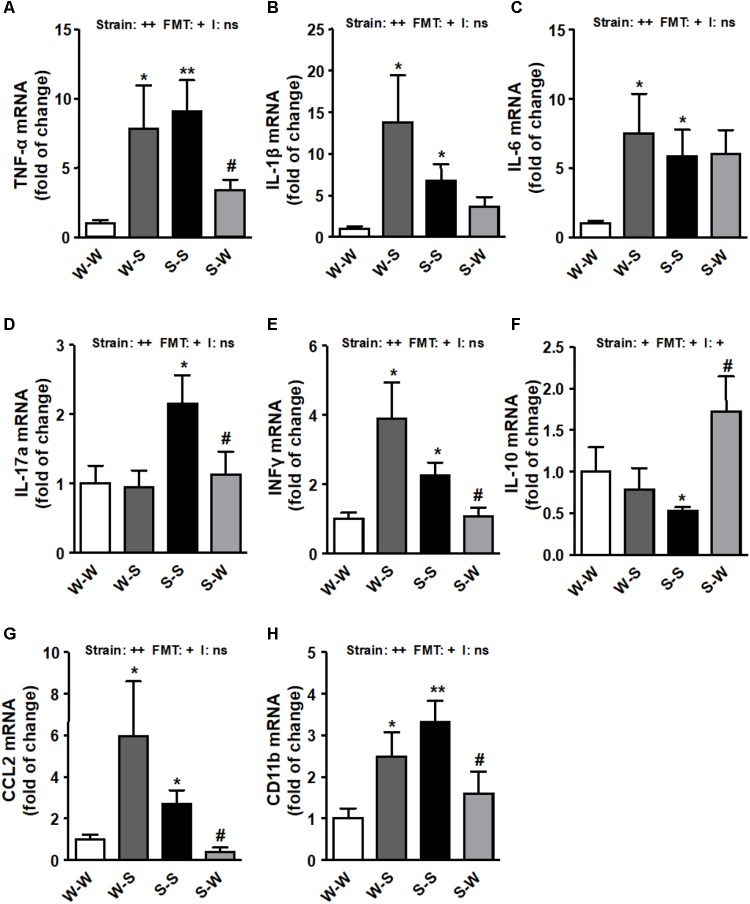
Effects of fecal microbiota transplantation (FMT) on brain PVN pro-inflammatory markers expression. mRNA levels of TNF-α **(A)**, IL-β **(B)**, IL-6 **(C)**, IL-17a **(D)**, interferon-γ (IFNγ) **(E)**, IL-10 **(F)**, C-C chemokine ligand 2 (CCL2) **(G)** and macrophage marker CD11b **(H)** measured by RT-PCR in brain PVN from all experimental groups. Strain factor, FMT factor and I interaction between strain and FMT factors. ++*p* < 0.01, +*p* < 0.05 and ns (not significant) for the probability based on a two-way analysis of variance. Values are means ± SEM (*n* = 5–8). ^∗^*P* < 0.05 and ^∗∗^*P* < 0.01 vs. WKY with stool transplant from WKY (W-W); ^#^*P* < 0.05 vs. SHR with stool transplant from SHR (S-S), statistical significance for the probability based on a Sidak’s correction multiple comparisons test.

We found that ROS production (*p*_strain_ < 0.01) (Figure 3A), NADPH oxidase activity (*p*_strain_ < 0.01) (Figure 3B) and the mRNA levels of NADPH oxidase subunits, NOX-1, NOX-4, p47^phox^, and p22^phox^ (*p*_strain_ < 0.01) (Figure 3C) and pro-inflammatory cytokines (tumor necrosis factor-α (TNF-α), interleukin (IL)-1β, IL-6, IL-17a, and interferon (IFN)-γ (*p*_strain_ < 0.01) (Figure 4A–E) in brain PVN were higher in the S-S group than those found in the W-W group, and were reduced by FMT from WKY rats to SHR (*p*_FMT_ < 0.05). However, these patterns did not led to a significant strain versus FMT interaction. By contrast, the anti-inflammatory cytokine IL-10 was reduced in the S-S group as compared to the W-W group (*p*_strain_ < 0.05) and increased after FMT from WKY to SHR (*p*_FMT_ < 0.05) (Figure 4F) leading to a significant strain versus FMT interaction (pi < 0.05). In addition, the mRNA levels of CCL2 (*p*_strain_ < 0.05) (Figure 4G) and CD11b (*p*_strain_ < 0.01) (Figure 4H) were also increased after FMT from SHR to WKY and decreased after FMT from WKY to SHR (*p*_FMT_< 0.05), without significant interaction (pi = 0.10).

**FIGURE 5:**
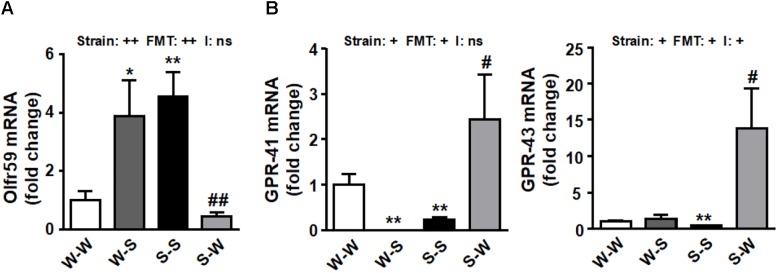
Effects of fecal microbiota transplantation (FMT) on brain PVN SCFA-sensing receptors expression. mRNA levels of Olfr 59 **(A)**, GPR-41 and GPR-43 **(B)** measured by RT-PCR in brain PVN from all experimental groups. Strain factor, FMT factor and I interaction between strain and FMT factors. ++*p* < 0.01, +*p* < 0.05 and ns (not significant) for the probability based on a two-way analysis of variance. Values are means ± SEM (*n* = 5–8). ^∗^*P* < 0.05 and ^∗∗^*P* < 0.01 vs. WKY with stool transplant from WKY (W-W); ^#^*P* < 0.05 and ^##^*P* < 0.01 vs. SHR with stool transplant from SHR (S-S), statistical significance for the probability based on a Sidak’s correction multiple comparisons test.

We sought to determine whether there were alterations in the genetic expression of olfactory receptors at the PVN. Interestingly, we found an increase of Olfr59 (*p*_strain_ < 0.01) in PVN accompanied by a downregulation of GPR-41 and GPR-43 (*p*_strain_ < 0.05) (Figure 5A,B) in SHR as compared to the W-W group. FMT from WKY to SHR restored the mRNA levels of these receptors (*p*_FMT_ < 0.01, pi = 0.37; *p*_FMT_< 0.05, pi = 0.08; *p*_FMT_< 0.05, pi < 0.03, respectively) (Figure 5A,B).

### FMT Induced Changes in the Gut Microbiota Composition

**FIGURE 6:**
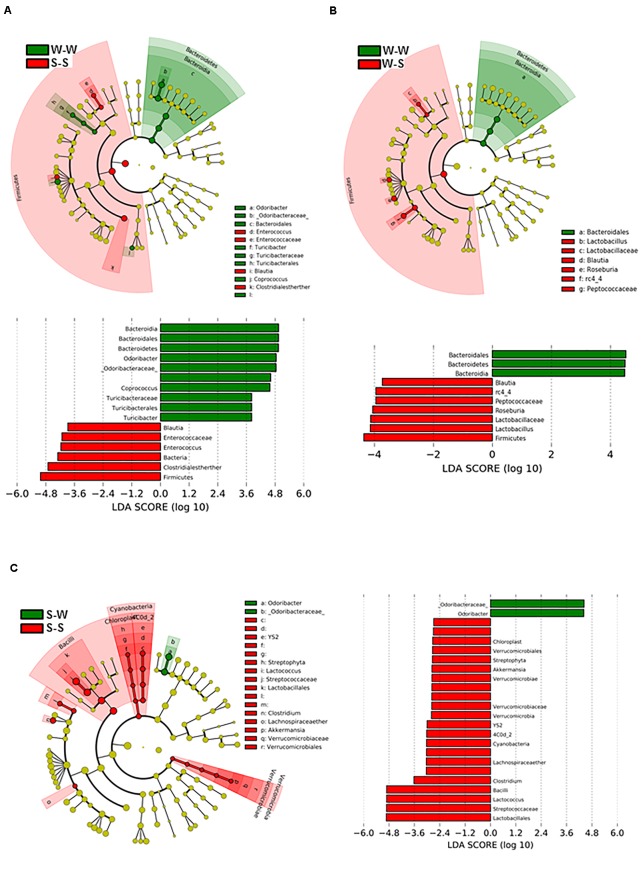
Effects of fecal microbiota transplantation (FMT) on changes of the gut microbiome. Comparisons of microbiome changes in WKY with stool transplant from WKY versus SHR with stool transplant from SHR **(A)** WKY-WKY versus WKY-SHR **(B)** and SHR-SHR versus SHR- WKY **(C)**. Cladograms (top panes) show the significantly enriched taxa, the taxa are identified in the key to the right of each pane. The larger the circles the greater the difference in abundance between the groups. The lower panels show the results of linear discriminant analysis effect size at *p* < 0.05 and LDA score of > 2.5 and detail the taxa most enriched by WKY, SHR or SHR-WKY. *n* = 6 animals per treatment group in each comparison.

To determine the dynamics of gut microbiota during the exchange of gut microbiota between SHR and WKY, we analyzed fecal DNA isolated from all experimental groups. Figure 6A shows the bacterial taxa (class, order, family, and genus) that were altered by fecal exchange from SHR to WKY, according to LEfSe analysis. Prominent shifts in bacterial community were observed after 4 weeks of treatment between the W-W and S-S groups, with an increase in the relative abundance of 10 bacterial taxa (green) and a decreasing of 6 taxa (red) comparing with the W-W group. Several changes in microbial taxa were also driven by fecal exchange from SHR to WKY with an increase in the relative abundance of 3 bacterial taxa (green) and a reduction of 7 taxa (red) (Figure 6B). While S-W compared to the S-S group, only relative abundance of 2 taxa were increased (green) and 22 were decreased (red) (Figure 6C). Interestingly, we found an association between a major abundance of butyrate-producing family *Odoribactereae* and the genus of *Odoribacter* with low BP levels present in the W-W and S-W groups. In contrast, we observed a correlation between *Blautia* and *Peptococcaceae* abundance and elevated BP in the S-S and W-S groups (Figure 6).

**FIGURE 7:**
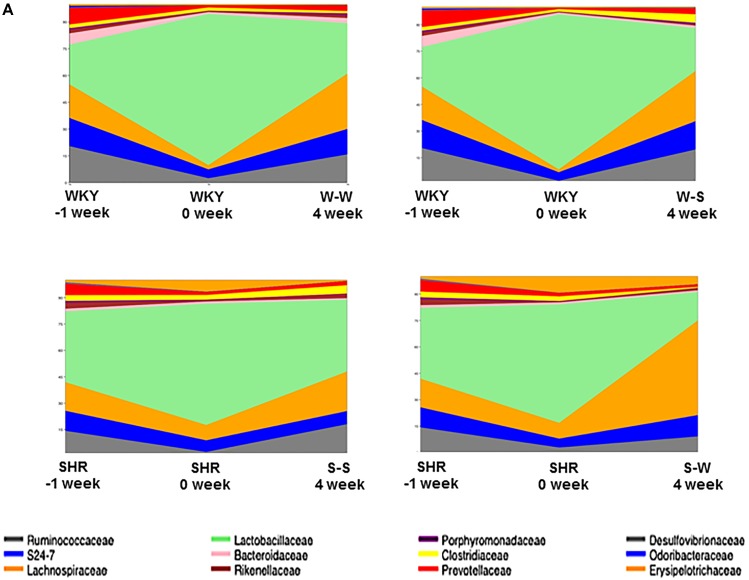
Effects of fecal microbiota transplantation (FMT) on changes of composition of gut microbiota. Time-dependent changes of fecal microbiota upon in all experimental groups (*n* = 6 rats per group).

When comparing the bacterial composition evolution, at the family level, in the gut microbiota between all experimental groups, we found that W-S had a significantly lower abundance of *Bacteroidaceae*, and greater abundance of *Clostridiacea* in the gut microbiota than the W-W group at 4 weeks of treatment. While S-W showed a depletion of abundance of *Lactobacillales* and an increase of *Erysipelotrichaceae* compared to the S-S group at the end of the experiment (Figure 7).

### Gut Integrity and Inflammation Are Regulated by Gut Microbiota

**FIGURE 8:**
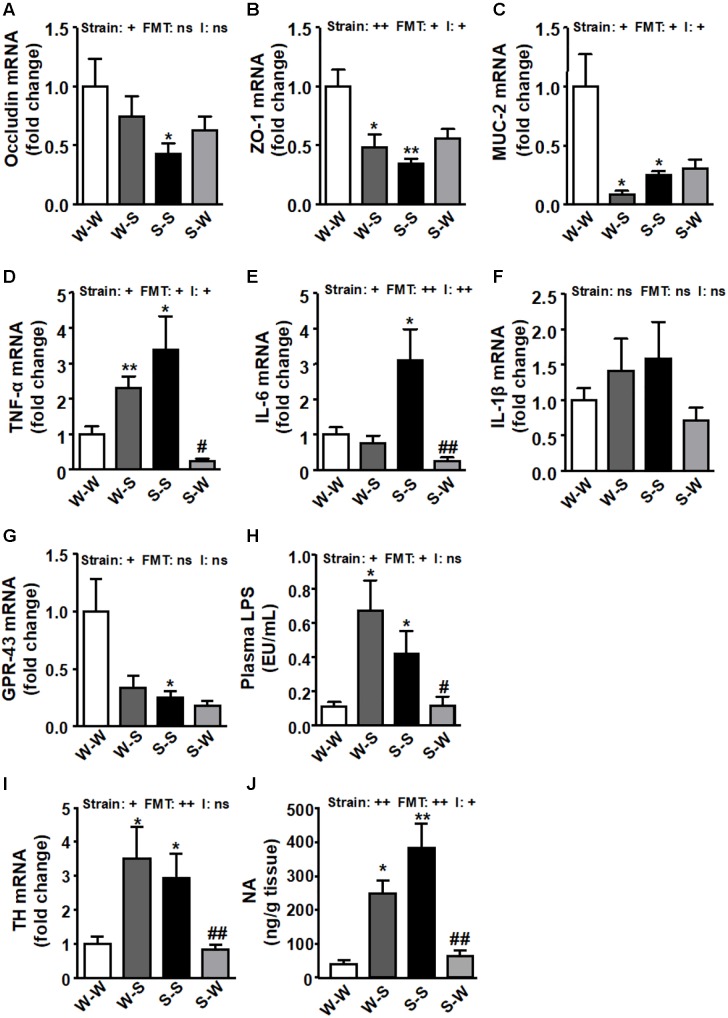
Effects of fecal microbiota transplantation (FMT) on colonic pro-inflammatory, epithelial integrity, and sympathetic activity markers. Colonic occludin **(A)**, zonula occludens-1 (ZO-1) **(B)**, mucin (MUC)-2 **(C)**, TNF-α **(D)**, IL-6 **(E)**, IL-β **(F)** and GPR-43 **(G)** mRNA levels, plasma endotoxin concentrations (EU/mL, endotoxin units/mL) **(H)**, mRNA levels of tyrosine hydroxylase (TH) **(I)**, and noradrenaline (NA) content **(J)**, in all experimental groups. Strain factor, FMT factor and I interaction between strain and FMT factors. ++*p* < 0.01, +*p* < 0.05 and ns (not significant) for the probability based on a two-way analysis of variance. Values are means ± SEM (*n* = 5–8). ^∗^*P* < 0.05 and ^∗∗^*P* < 0.01 vs. Wistar-Kyoto (WKY) with stool transplant from WKY (W-W); ^#^*P* < 0.05 and ^##^*P* < 0.01 vs. SHR with stool transplant from SHR (S-S), statistical significance for the probability based on a Sidak’s correction multiple comparisons test.

In concurrence with previous data (Santisteban et al., 2017) the levels of occludin (*p*_strain_ < 0.05) (Figure 8A), and zonula occludens (ZO)-1 (*p*_strain_ < 0.01) (Figure 8B), and mucin (MUC)-2 (*p*_strain_ < 0.05) (Figure 8C) were significantly reduced in the S-S group as compared to the W-W group. The expression of colonic ZO-1 and MUC-2 (*p*_FMT_ < 0.05) were also reduced when FMT from SHR to WKY was performed, which were accompanied of a significant strain versus FMT interaction (pi < 0.05), whereas FMT from WKY to SHR tended to increase these parameters but without statistical significance, as compared to the S-S group. Similarly, increased mRNA expression of pro-inflammatory TNF-α (*p*_strain_ < 0.05) (Figure 8D) and IL-6 (*p*_strain_ < 0.05) (Figure 8E), without significant change in IL-1β (Figure 8F) was also observed in the S-S group as compared to the W-W group. FMT from WKY to SHR reduced the levels of these pro-inflammatory cytokines (*p*_FMT_< 0.05, pi < 0.05; *p*_FMT_< 0.01, pi < 0.01, respectively). We found reduced GPR-43 mRNA levels in the gut from both SHR groups as compared to the W-W group (*p*_strain_ < 0.05) (Figure 8G). GPR-43 transcript level tended to be reduced in WKY rats after FMT from SHR (*p*_FMT_ = 0.057) without interaction between strain and FMT (pi = 0.11). In addition, an increase in intestinal permeability has been proposed as a crucial mechanism for the development of endotoxemia (Cani et al., 2008). In fact, we found increased plasma LPS levels Figure 8H) in rats with high BP (W-S and S-S groups) (*p*_strain_ < 0.05; *p*_FMT_ < 0.05; pi = 0.19), qualitatively associated with impaired colonic integrity. Interestingly, FMT transplantation from WKY to SHR reduced plasma LPS levels despite no significant increase in gut integrity. Furthermore, both the colonic expression of tyrosine hydroxylase (TH) (*p*_strain_ < 0.05) (Figure 8I) and the colonic NA concentration (*p*_strain_ < 0.01) (Figure 8J) were significantly up-regulated in both groups that received fecal contents from SHR. Interestingly, the long-term treatment with stool from WKY significantly decreased both TH levels (*p*_FMT_< 0.01; pi = 0.62) and NA content (*p*_FMT_ < 0.01; pi < 0.05) in the S-W group.

## Discussion

Fecal transplantation from animals (Adnan et al., 2017; Toral et al., 2018) and subjects (Li et al., 2017) with hypertension to normotensive animals can elevate BP. However, the mechanisms by which bacteria control BP have not been elucidated. Our results demonstrate, for the first time, that alteration of gut microbiota composition induced by reciprocal FMT between WKY and SHR influences the brain, and SNS impacting BP. The most significant findings of this study are; (1) Microbiota affects brain PVN NADPH oxidase activity, neuroinflammation and sympathetic activity in both strain of rats, (2) Lower *Blautia* and *Odoribacter* content in feces is inversely correlated with high SBP, and (3) Loss of gut integrity in SHR seems to be independent of sympathetic tone, BP, and microbiota composition.

It is pertinent to note that elevated SNS activity is a hallmark of both animal and human hypertension (DiBona, 2013; Grassi et al., 2015). Santisteban et al. (2017) observed enhanced gut-neuronal communication in hypertension originating from the PVN of the hypothalamus and presenting as increased sympathetic drive to the gut. Previous studies have also characterized hypertension with alterations in the gut microbiota and sympathetic dysregulation (Zubcevic et al., 2014; Yang et al., 2015; Santisteban et al., 2017). Our current study, demonstrating both higher BP reductions after pentolinium administration and plasma NA levels, both markers of increased sympathetic drive, associated with microbial dysbiosis, provides new evidence in support of that proposal. This higher SNS activity correlates with higher SBP and DBP in the W-S group compared to W-W. Similarly, FMT from SHR to SHR showed higher levels of these sympathetic drive parameters than that found in the W-W group. The changes induced by FMT from WKY to SHR and vice versa in BP seem to be independent of systemic renin-angiotensin system, since PRA was not significantly affected. Moreover, increased catecholaminergic neurotransmission has been reported in SHR, characterized by increased TH activity as well as gene and protein expression (Yu et al., 1996; Reja et al., 2002; Lopez Verrilli et al., 2009), suggesting that TH plays a key role in the genesis, development and/or maintenance of hypertension. In agreement with this information, our data showed increased colonic expression of TH and NA content from the S-S and W-S groups compared to W-W, showing increased sympathetic activity in this tissue.

The gut microbiota influences the hosts inflammatory response (Cani et al., 2007) and inflammation induces oxidative stress and vice versa. In brain, angiotensin II via an AT1 receptor mechanism activates the sympathetic outflow by stimulation of the NADPH oxidase-dependent ROS production (Gao et al., 2005). In brain from the S-S group we found increased NADPH oxidase activity driven-ROS production, expression of NADPH oxidase subunits, pro-inflammatory cytokines (TNF-α, IL-1β, and IL-6) and sympathetic activity, as compared to W-W rats. These data could be related to higher PRA found in the S-S group as compared to the W-W group. Conversely, FMT from SHR to WKY also resulted in higher PVN inflammation, NADPH-oxidase activity and sympathetic outflow than induced by FMT from WKY to WKY. Taken together, our data suggest that gut microbiota is involved in the regulation of brain inflammatory and oxidative status, and the subsequent sympathetic activity.

The increased sympathetic activity also affects the BM resulting in an increase in inflammatory cells, which migrate to the PVN and enhance neuroinflammation (Santisteban et al., 2016). Accordingly, we found increased inflammation in brain PVN from the S-S and W-S groups, associated to an increased expression of CCL2, which facilitates BM cells entering the brain’s parenchymal space; CD11b, a macrophage marker; and IL-17a, mainly produced by Th17 cells, that might contribute to neuroinflammation. Overall, all this data showed a gut-brain communication characterized by increased pro-oxidant, pro-inflammatory and immune cell infiltration profile in brain PVN after FMT from SHR to WKY. Interestingly, chronic normal microbiota transplantation to hypertensive rats induced a stable BP reduction linked to reduced brain PVN inflammation, NADPH oxidase activity and sympathetic excitation. Taken together, our present results demonstrate that hypertension is, at least in part, a result of pathophysiological changes in the gut microbiota, affecting brain areas of cardiovascular control such as PVN.

The mechanisms involved in the pro-hypertensive effects of gut microbiota from SHR are unknown. There is growing evidence that gut microbiota has emerged as an important factor that can influence the host’s physiology through bacterial metabolic products such as SCFAs (Pluznick et al., 2013). Gut dysbiosis in SHR is characterized by reduced acetate- and butyrate-producing bacteria than their WKY normotensive counterparts (Yang et al., 2015). These SCFAs induced anti-inflammatory effects in the gut, mediated by GPR-43 activation (Yang et al., 2018). SCFAs, in addition to providing energy to gut epithelium and peripheral tissues, also promote intestinal epithelial integrity and aid in the repair of wounded epithelium, mainly throught GPR-43 activation (D’Souza et al., 2017). We can hypothesize that insufficient signaling through SCFAs-activated GPR-43 pathway in the gut, mediated by both lower SCFAs-producing bacteria and lower colonic GPR-43 expression in SHR, can lead to compromised gut integrity, dysregulated inflammation, and passage of substances such as LPS into the blood. The increase in BP in SHR was associated with gut pathology that included increased intestinal permeability and decreased tight junction proteins (Santisteban et al., 2017). We analyzed the integrity of the gut epithelial barrier measuring the mRNA levels for tight junction proteins and mucins, which are involved in mucus production, in the proximal colon. We found that FMT from SHR to WKY significantly reduced colonic ZO-1 and MUC-2, increased colonic TNF-α and plasma levels of LPS. LPS might be involved in the pathogenesis of hypertension, through toll-like receptor (TLR)-4 stimulation in the vasculature (Liang et al., 2013), and by inducing systemic inflammation, accompanied by microglia activation, oxidative stress in cardiovascular regions of the brain, such as rostral ventrolateral medulla (Wu et al., 2012) and PVN (Zhang et al., 2010). However, high levels of plasmatic LPS, as a consequence of an infection, can lead to septic shock and hypotension (Bermejo et al., 2003).

On the other hand, butyrate, acetate and propionate are metabolic products of the gut bacteria that, in addition to their gut anti-inflammatory effects, have anti-hypertensive properties (Natarajan and Pluznick, 2014; Pluznick, 2014; Natarajan et al., 2016). SCFAs modulate BP through the renal and vascular Olfr and GPRs. SCFAs modulate BP through the renal and vascular olfactory receptor (Olfr) 59 (its stimulation elevates BP), G-protein-coupled receptor (GPR)-41, and GPR-43 (their stimulation both lower BP) (Pluznick et al., 2013). In addition to the vasculature (Pluznick et al., 2013; Pluznick, 2014), the SCFA-sensing receptors Olfr78 and GPR-43, as well as GPR-41, are present on the sympathetic ganglia (Kimura et al., 2011; Nøhr et al., 2015). We also found expressional changes in PVN of the hypothalamus (a major brain region involved in regulation of the sympathetic output) of receptors for SCFAs, suggesting a probable connection with BP control. Prior works have demonstrated that a high abundance of butyrate-producing genus *Odoribacter*, in the gut microbiota in pregnant women at 16 weeks gestation is associated with decreased BP (Gomez-Arango et al., 2016). As butyrate is able to cross the blood brain barrier (Bergersen et al., 2002; Vijay and Morris, 2014; Sun et al., 2016) circulating butyrate may thus have direct effects on regions of the brain that regulate BP. Our data showed that a significant depletion of *Odoribacter* in the W-S and S-S groups were noted, consequently, this depletion might contribute to the dysregulation of the expression Olfr59, GPR-41, and GPR-43 found in PVN from hypertensive groups. Because Olfr59 elevates BP and GPR-41 opposes this action to lower BP, these data support the suggestion that the altered expression of SCFA receptors in the PVN may play a role in elevated BP of the SHR. Several groups have reported the presence of SCFA-sensing receptors (i.e., Olfr59, GPR-41) in multiple organs and neural tissues (Kimura et al., 2011; Nøhr et al., 2015). These studies also show that expression levels of SCFA-sensing receptors in the whole brain are relatively low (Kimura et al., 2011), but no studies to date have specifically examined the expression of these receptors in cardioregulatory brain regions such as the PVN of the hypothalamus. Moreover, the effects of SCFAs on modulation of sympathetic activity and BP have also previously been suggested. Kimura et al. (2011) observed that intraperitoneal administration of propionate caused a significant increase in heart rate in the wild type, but not the Gpr41-/- mice. It is tempting to hypothesize that the possible low plasma butyrate levels and lower GPR-41, and GPR-43 expression found in PVN from rats transplanted with SHR microbiota as compared to that transplanted with WKY feces, could also contribute to increased neuroinflammation and sympathetic outflow in these animals. However, neither plasma nor brain levels of SCFAs have been measured in the present study, which limit the real role of SCFAs in the control of sympathetic output. Clearly, additional studies are necessary to confirm this hypothesis and provide a mechanism for the differential regulation of these SCFA receptors throughout the SNS.

Furthermore, we found in the W-S group a depletion of *Peptococcaceae*, a sulfate-reducing bacteria considered to be beneficial in the regulation of BP (Ahmad et al., 2012; Yu et al., 2018). Sulfate-reducing bacteria, and hydrogen sulfide released in the colon may also contribute to the control of arterial BP, being antihypertensive, at least in part, through suppression of sympathetic outflow (Duan et al., 2015). The genus *Blautia*, which is the main bacterial group in *Clostridium* coccoides-group, was also found to decrease in Japanese patients with type 2 diabetes, as compared with control subjects (Sato et al., 2014).

Interestingly, transplantation of gut microbiota from WKY to SHR reduced gut inflammation, plasma LPS levels, brain PVN inflammation, NADPH oxidase activity and sympathetic excitation, leading to a stable drop of BP. However, no significant improvement of colonic integrity was found in this group as compared to the S-S group. Sympathetic activity influenced these gut parameters (ZO-1, occludin, MUC-2), via top-down signaling (Santisteban et al., 2017). However, normalized sympathetic activity to the gut (lower TH transcript level and NA content) without correction of gut parameters, in the SHR with a WKY microbiota, is in conflict with this hypothesis. The lack of protective effects in the gut integrity could be related to the lower expression of GRP-43 found in colonic tissue from this group, and subsequent lower GRP-43 pathway activation despite the possible increase in gut SCFAs induced by FMT from WKY. A similar loss of gut integrity has been found in young prehypertensive SHR despite no gut dysbiosis being detected in these animals (Santisteban et al., 2017). Overall, gut integrity in SHR seems to be independent of sympathetic tone, BP, and microbiota composition.

## Conclusion

In conclusion, in this genetic model of hypertension (SHR) gut dysbiosis correlates to sympathetic outflow via stimulation of NADPH-oxidase-derived ROS in the brain. These central effects seem to be associated with reduced expression of butyrate-sensing receptors in the hypothalamus, Th17 and macrophages infiltration in PVN and higher plasma levels of LPS. These results are congruent with evidence from previous studies suggesting a strong correlation between hypertension and gut microbiota dysbiosis, establishing a cause-effect relationship between elevated BP and altered gut microbiota. We can speculate that changes in gut microbiota through the use of probiotics in treating gut dysbiosis could have positive effects on neurogenic hypertension, through modulation of central sympathetic activity.

## Author Contributions

MT and JD participated in the research design. MT, IR-V, NdlV, MR, MS, MG-G, and RJ performed most of the experiments. IR-V, TY, and MR performed the 16S rDNA V4–V5 region sequencing and bioinformatics analysis. MT, IR-V, MR, NdlV, RJ, and JD analyzed the data. MT and JD wrote or contributed to the writing of the manuscript. All authors approved the final version of the manuscript to be published.

## Conflict of Interest Statement

The authors declare that the research was conducted in the absence of any commercial or financial relationships that could be construed as a potential conflict of interest.

## Abbreviations

BM: bone marrow
BP: blood pressure
DBP: diastolic blood pressure
DHE: dihydroethidium
DNA: deoxyribonucleic acid
FMT: fecal microbiota transplantation
GPR: G-protein-coupled receptor
HR: heart rate
IFN: interferon
IL: interleukin
LPS: lipopolysaccharide
MUC: mucin
NA: noradrenaline
O_2_^-^: superoxide anion
of TH: tyrosine hydroxylase
Olfr: olfactory receptor
PRA: plasma renin activity
PVN: paraventricular nucleus
ROS: reactive oxygen species
RT-PCR: reverse transcriptase-polymerase chain reaction
SBP: systolic blood pressure
SCFAs: short-chain fatty acids
SHR: spontaneously hypertensive rats
SNS: sympathetic nervous system
TLR: toll-like receptor
TNF-α: tumor necrosis factor-α
WKY: Wistar-Kyoto
ZO: zonula occludens
